# Androstenol – a Steroid Derived Odor Activates the Hypothalamus in Women

**DOI:** 10.1371/journal.pone.0008651

**Published:** 2010-02-17

**Authors:** Ivanka Savic, Hans Berglund

**Affiliations:** 1 Stockholm Brain Institute, Karolinska Institutet, Department of Clinical Neuroscience, Stockholm, Sweden; 2 Department of Medicine, Karolinska University Hospital, Stockholm, Sweden; Duke University, United States of America

## Abstract

**Background:**

Whether pheromone signaling exists in humans is still a matter of intense discussion. In the present study we tested if smelling of Androstenol, a steroid produced by the human body and reported to affect human behavior, may elicit cerebral activation. A further issue was to evaluate whether the pattern of activation resembles the pattern of common odors.

**Methodology:**

PET measurements of regional cerebral blood flow (rCBF) were conducted in 16 healthy heterosexual women during passive smelling of Androstenol, four ordinary odors (OO), and odorless air (the base line condition).

**Principal findings:**

Smelling Androstenol caused activation of a portion of the hypothalamus, which according to animal data mediates the pheromone triggered mating behavior. Smelling of OO, on the other hand, engaged only the classical olfactory regions (the piriform cortex, lateral amygdala, anterior insular and anterior cingulate cortex).

**Conclusions:**

The observed pattern of activation is very similar to the pattern previously detected with 4,16-androstadien-3-one in heterosexual females. It suggests that several compounds released by human body may activate cerebral networks involved in human reproduction.

## Introduction

According to the original definition by Karlson and Lucher pheromones are chemical compounds secreted externally by some animals (especially insects) that influences the physiology or behavior of other animals of the same species [1]. Whilst pheromone effects in animals are well recognized, it is controversial whether they exist also in humans. Several psychophysical studies indicate that a progesterone derivative, 4,16-androstadien-3-one (AND) is capable of affecting mood, arousal, heart rate, as well as cortisol levels [2], [3], [4], [5], [6], [7]. Furthermore, recent brain imaging studies show that smelling of AND, as well as of an estrogen like compound, estra-1,3,5(10),16-tetraen-3-ol (EST), trigger cerebral activations suggestive of pheromone signaling [8], [9], [10], [11]. Sobel and Gabrieli [8] observed that smelling EST activates the thalamus, hypothalamus, and the prefrontal cortex in men even when this steroid was presented in non-consciously detectable concentrations. In a series of imaging studies employing EST and AND, we found activations in the anterior hypothalamus, which were differentiated with respect to sex and sexual orientation of the smeller in relation to the respective compound [9], [10], [11]. Heterosexual women and homosexual men displayed activations of the hypothalamus when smelling AND, whereas smelling of EST recruited classical olfactory regions (the amygdala, piriform, anterior cingulate and insular cortex). The pattern of activation was reciprocal in heterosexual men and homosexual women [9], [10], [11]. Considering that the anterior hypothalamus is the major mediator of pheromone signals, these data strongly argue that such signals may exist also in humans. In order for this concept to be valid, however, several bodily released compounds should have pheromone properties. Gas chromatographic analyses of human body odors suggest that in addition to complex mixtures of aliphatic carboxylic acids, volatile steroids are prime candidates to serve pheromone function (Gowern, Ruparella 1993). Androstenol (5a-androst-16-en-3a-ol) is a steroid, which like AND belongs to the group of odorous 16-androstenes. Androstenol was first isolated from boar testes, and several animal experiments suggest that androstenol is capable of reducing anxiety, as well as hippocampal epileptogenic activity [12]. It was subsequently detected in humans, (primarily in males), in sweat, urine, plasma and saliva [13]. Androstenol is also shown to affect hormonal, behavioral and social responses in humans [14], [15]. Of particular note is its influence on the pulsatile secretion of luteinizing hormone in human females, an effect assumed to be mediated by the hypothalamic nuclei [16]. Considering that this compound also is highly volatile (vapor pressure at 37°C is 254 microtorr), it could be expected to act as a pheromone, provided that its signals can be transduced to the brain. To test this possibility, we carried out activation studies with positron emission tomography (PET) in which we measured changes in cerebral blood flow during passive smelling of Androstenol compared to smelling unscented air. Two issues were addressed in particular:

1. Does smelling of Androstenol activate the human brain?
2. Does the site of activation correspond to activations previously detected with ordinary odors?

## Methods

Fifteen right-handed, healthy, unmedicated, heterosexual women (age 22–38 years) were investigated during the second to third week of the menstrual cycle. None of the subjects had allergy, upper respiratory problems, or heredity for anosmia. Neither did they have history of or heredity for neuropsychiatric disorders. Their olfactory thresholds, measured prior to the PET study with n-butyl alcohol as described previously[9], was normal (9×10^−5^±3×10^−5^ M).

The study was approved by the Ethics and Radiosafety Committees at the Karolinska Institute.

### PET Experiments

Regional cerebral blood flow (rCBF) was measured during baseline and activation using ^15^O-H_2_O-PET. During base line (denoted AIR in the manuscript) subjects were laying in the scanner with closed eyes, plugged ears, passively breathing of the unscented environmental air. There were two activation conditions: 1) birhinal smelling of Androstenol, 2) birhinal smelling of four different odors, which were presented on line during the same scan and denoted as OO throughout the manuscript. The rationale for presenting four odors during the same scan was to avoid that results would rely on one odor, but also avoid an excessive radioactivity exposition which would be a consequence of separate scans for the separate odors. The odors were butanol, cedar oil, lavendel oil and eugenol. Butanol was diluted in distilled water (10% concentration), the other odors were not diluted. Psychophysical characteristics of the four odors have been described in our earlier studies, which showed reproducible activations of the amygdala, piriform cortex, and portions of the anterior insular and cingulate cortices, in accordance with other studies of odor activation [17], [18], [19], [20]. These odors were used to investigate whether Androstenol engages similar cerebral regions as the more ordinary odors. Because, theoretically, dissolving of Androstenol could change its possible pheromone properties we chose to present it in crystalline and odorous form (200 mg, Steraloids, Newport, RI). The purity was 98%, as tested repetitively at our doping laboratory (Department of Pharmacology, Karolinska University Hospital).

PET experiments were carried out as described previously [9]. The room temperature and air pressure were standardized (23°C, 997 hPa) [9]. Subjects were informed that they would smell either odor or unscented air while an open and empty, or odor containing jar was presented 10 mm under the nose, four times during 15 seconds with 5 seconds breathing of environmental air in-between [9], [10], [11]. Subjects were instructed to relax, with eyes closed and ears plugged, and just breathe normally without sniffing or hyperventilating (which they trained twice before the PET sessions). There were six PET scans per person, lasting 60 seconds (three conditions, two scans/condition, balanced and randomly interleaved). Respiratory movements were recorded continuously 2 min before, and during each scan, by using a strain gauge around the lower thorax connected to a graph (Comair, Stockholm, Sweden) as described previously [9]. When all the PET scans were completed subjects were presented with Androstenol and OO again and asked to rate each item for odor intensity, irritability, pleasantness, and familiarity, using a Visual Analogue Scale (VAS), [9], [11], [18]. Differences in ratings between groups were tested with paired T-tests, one for each modality. The mean respiratory amplitude and the frequency was first calculated during each prescan and scan period. The baseline and scan-associated amplitude and frequency were stable within the same subject throughout the study but tended to vary from subject to subject. Rather than carrying out comparisons on the basis of absolute values during various conditions, we, therefore, analyzed the relative change in respiratory amplitude and frequency during each presentation by calculating the mean percentage difference between the scan- and immediate prescan values. Mean relative change in respiratory amplitude and frequency was compared between AIR, Androstenol and OO by means of repeated measures ANOVA. The significance level was 0.05 in all comparisons.

Activations and deactivations were calculated with SPM2 software package (Wellcome Department of Cognitive Neurology, London, http://www.fil.ion.ucl.ac.uk/spm). Activations were defined contrasting Androstenol-AIR and OO-AIR, deactivations running the contrasts in the opposite direction (AIR – Androstenol and AIR–OO). Congruent with several of our previous publications with odor stimuli the T-threshold was calculated at *P* = 0.01, corrected *P*<0.05 (one group random effect analysis).

## Results

**Figure 1:**
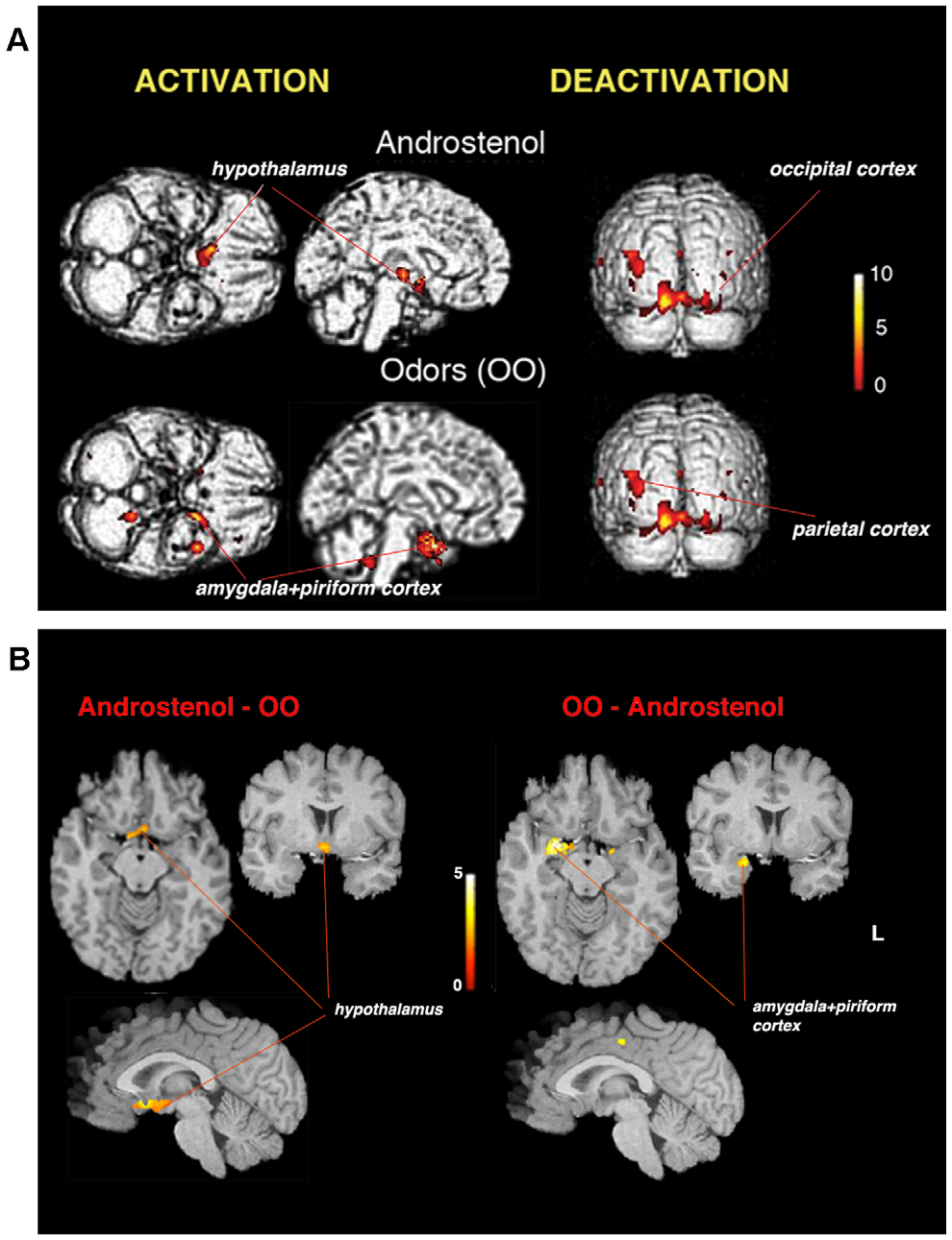
Activations and deactivations with androstenol and OO. Upper: Activation and deactivation with Androstenol and OO**.** Activation (left) and deactivation (right) detected during smelling of Androstenol, and odors (OO). The OO cluster is located in the amygdala and piriform cortex. Lower: Difference between activations with Androstenol and odors (OO). The Frontal lobe clusters in the Androstenol – OO contrast were not significant. Clusters calculated at *P* = 0.01, corrected *P*<0.05 are superimposed on the standard brain MRI. The Sokoloff's color scale illustrates *T* values reflecting the degree of activation.

**Figure 2:**
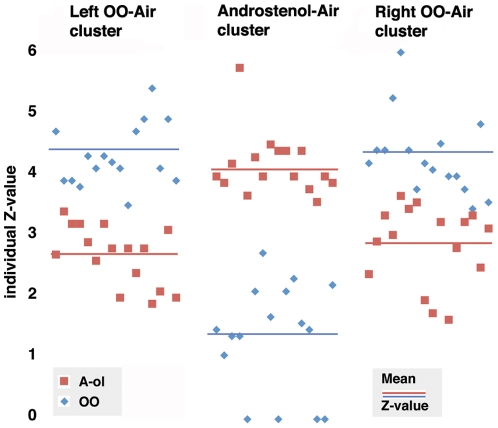
Individual Z-values in the activated clusters. Graphical presentation of individual Z-values from Talairach's co-ordinate 4 0 -18 (peak activation in the hypothalamus cluster derived from Androstenol – Air, and the Talairach's coordinates 18 -10 -4 and -22 2 -8 (peak activations in the right and left amygdala+piriform cortex clusters derived from OO - Air. The individual values were obtained from the respective peak co-ordinate by lowering the uncorrected threshold to *P* = 0.1, which allowed retrieval of the respective Z-values although they were far below the significance level for the respective contrast (*P* = 0.01, corrected p<0.05, please see the main text). The figure illustrates that that the hypothalamus networks were recruited during smelling of Androstenol, but not OO. Vertical line indicates Z-values, horizontal line the individual subjects).

As shown previously, smelling of OO caused activation of the amygdala, piriform, anterior insular cortex, and portions of the anterior cingulate cortex. In contrast, smelling of Androstenol yielded activations in the anterior hypothalamus and the medial portion of the right amygdala (Table 1, Figure 1, 2). When lowering the corrected *P*-value to 0.1, additional cluster appeared in the left amygdala and piriform cortex, which is congruent with the notion that the participating subjects perceived Androstenol as odorous. Deactivations were detected in the parietal and temporal neocortex with both OO and Androstenol, and were more pronounced with OO. When comparing activations caused by OO and Androstenol, a significant cluster was detected in the right piriform cortex, (Talairach's co-ordinates 22 14 -18, cluster size 1.3 cc, Z = 4.6 for OO - Androstenol, and -4 1 -5, cluster size 0.9 cc, Z = 3.9 for Androstenol – OO, Figure 1).

**Table 1:** Activations and deactivations with Androstenol and OO.

Region	Z level	Size, cm^3^	Co- ordinates	Z level	Size, cm^3^	Co- ordinates
**Activation**	**Androstenol - AIR**			**OO - AIR**		
R Amygdala and piriform cortex				4.9	10.4	18 -10 -4
				4.5	10.1	-22 2 -8
Anterior hypothalamus and R medial amygdala	3.4	3.6	4 0 -18			
**Deactivation**	**AIR - Androstenol**			**AIR - OO**		
L Temporal neocortex				4.3	6.0	-58 -54 -2
L, R Parieto-occipital cortex	4.7	1.4	-10 -88 18	4.4	4.0	-10 -84 -6 42 -68 18
Temporal neocortex	6.2	6.4	50 -48 -10 28 -66 0	4.0 4.2	3.0 4.0	34 -64 0 -12 -74 40

**General note:** Values calculated using SPM2, height threshold at *P* = 0.01, corrected *P*<0.05.

Talairach's co-ordinates indicate peak activation; the indicated regions describe coverage of the respective cluster. L  =  left; R  =  right.

Both OO and Androstenol were perceived as fairly neutral (scoring around 50 mm on the Visual Analogue Scale) with respect to odor familiarity, irritability and pleasantness, (Table 2). The VAS scores for odor intensity were, however, significantly higher for OO (7.3±1.3 vs. 4.4±2.1; paired T-test, *P* = 0.009). No significant difference was found in the change in respiratory amplitude (F = 2.2, p = 0.13) or frequency (F = 0.10, *P* = 0.90) detected during presentation of Androstenol, OO, or AIR (Table 3).

**Table 2:** Odor ratings.

	Pleasantness	Intensity	Irritability	Familiarity
Androstenol	42±25	44±21	29±28	49±21
OO	44±17	72±13**	42±25	54±12

**p = 0.009; the other comparisons in odor ratings were not significant (paired t-test, *P*<0.05). OO ratings represent means of ratings for butanol, cedar oil, lavendel oil and eugenol. The values (mm), expressed as mean and standard deviation, were generated using Visual Analogue Scale.

**Table 3:** Respiratory change in amplitude and frequency.

	Air (%)	Androstenol (%)	OO (%)	
Amplitude	6.6±30.1	30.7±43.2	22.9±45.8	F = 2.2, *P* = 0.13
Frequency	8.7±10−7	10.0±14.5	7.8±16.0	F = 0.10, *P* = 0.90

Values are expressed as mean and standard deviation in the % change of respiratory frequency and amplitude during scan- vs. prescan-recordings.

## Discussion

Androstenol is a bodily released volatile compound, which has been detected in male sweat. Although this compound is reported to evoke psychophysical response in humans [15], [16], its role as a potential human pheromone has been questioned in the absence of studies showing that our brains can detect signals elicited by smelling of this compound. The observed pattern of activation with Androstenol was very similar to the previously detected pattern in heterosexual women and homosexual men during smelling of AND [9], [10] [11]. This similarity is unlikely to be an effect of the steroid structure, considering that smelling of AND (and also EST) was found to elicit a dual pattern of activation recruiting primarily the hypothalamus or the olfactory regions (the amygdala, piriform and insular cortex), depending of the sex and sexual orientation of the smeller [9]. The present study shows that smelling of Androstenol and OO recruits different neuronal circuits (Figure 1 and 2). Androstenol was deemed to be a weaker odor than OO, (Table 2), suggesting that the OO-specific activation in the amygdala and piriform cortex may be a reflection of intensity. This may, however, not explain lack of hypothalamus activation by OO. Congruent with our previous discussions [9], [10] we, therefore, suggest that Androstenol, like AND and EST, may act bimodally, both as an odor and a pheromone-like compound, and that the hypothalamus activation is caused by a non-odor component (presumably a pheromone-like component), whereas the weaker activation of the medial amygdala and piriform cortex was due to the odor component.

Androstenol is secreted in sweat. When using human sweat as the stimulus, Lundstrom et al. detected significant clusters in the posterior cingulate cortex, the posterior occipital gyrus, the dorsal postcentral and angular gyrus, but not in the hypothalamus (15). The aim of that study was, however, to differentiate between activations by axillary secrete from kin and non-kin persons of the same sex, and the results were interpreted to reflect engagement of self-referential regions. The generated data were, thus, not directly comparable with the present (15). Furthermore, sweat contains various compounds with different properties and mutual interactions, which may lead to more complicated activation patterns. Nevertheless, it is of interest that both studies showed a major activation of non-olfactory regions, suggesting that components of human sweat may affect the human brain in an odor-unrelated manner. This allows us to conclude that passive smelling of several different 16-androstenes activates the brain of healthy heterosexual women, and that this activation differs from activations by ordinary odors. The finding that smelling of a previously untested compound shows a primary engagement of cerebral structures involved in human reproduction, although entirely new study group was investigated, motivates further investigations of human pheromone biology.
